# Novel *S. cerevisiae* Hybrid Synthetic Promoters Based on Foreign Core Promoter Sequences

**DOI:** 10.3390/ijms22115704

**Published:** 2021-05-27

**Authors:** Xiaofan Feng, Mario Andrea Marchisio

**Affiliations:** School of Pharmaceutical Science and Technology, Tianjin University, 92 Weijin Road, Tianjin 300072, China; fengxf95@tju.edu.cn

**Keywords:** synthetic biology, promoters, TATA box, UAS, gene expression, *Saccharomyces cerevisiae*

## Abstract

Promoters are fundamental components of synthetic gene circuits. They are DNA segments where transcription initiation takes place. New constitutive and regulated promoters are constantly engineered in order to meet the requirements for protein and RNA expression into different genetic networks. In this work, we constructed and optimized new synthetic constitutive promoters for the yeast *Saccharomyces cerevisiae*. We started from foreign (e.g., viral) core promoters as templates. They are, usually, unfunctional in yeast but can be activated by extending them with a short sequence, from the *CYC1* promoter, containing various transcription start sites (TSSs). Transcription was modulated by mutating the TATA box composition and varying its distance from the TSS. We found that gene expression is maximized when the TATA box has the form TATAAAA or TATATAA and lies between 30 and 70 nucleotides upstream of the TSS. Core promoters were turned into stronger promoters via the addition of a short UAS. In particular, the 40 nt bipartite UAS from the *GPD* promoter can enhance protein synthesis considerably when placed 150 nt upstream of the TATA box. Overall, we extended the pool of *S. cerevisiae* promoters with 59 new samples, the strongest overcoming the native *TEF2* promoter.

## 1. Introduction

*Saccharomyces cerevisiae*, the baker’s yeast, was the first eukaryote whose genome was completely sequenced [1]. Traditionally, *S. cerevisiae* has been used in brewing, winemaking, and baking [2]. More recently, with the advent of Synthetic Biology, *S. cerevisiae* has become a widely adopted chassis for genetic circuits due to its short growth cycle, simplicity of large-scale cultivation, and, above all, the easiness of genomic manipulation [3]. These properties have permitted turning *S. cerevisiae* cells into factories for the production of valuable chemicals such as drug precursors [4] and natural products [5].

**Figure 1:**
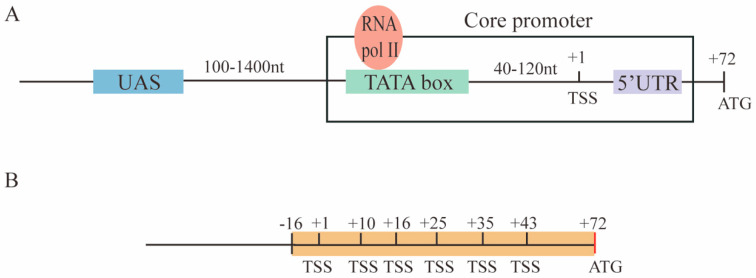
Promoters in *S. cerevisiae*. (**A**) The structure of promoters in the baker’s yeast is characterized by four main elements: The upstream activating sequence (UAS), the TATA box (where RNA polymerase II—RNA pol II—binds), the transcription start site (TSS), and the 5′UTR (untranslated region). The core promoter sequence consists of the TATA box(es), the TSS(s), and the 5′UTR. (**B**) The pCYC1noTATA begins at position −16 with respect to the TSS at position +1 in the *CYC1* yeast promoter. It contains six TSSs over 43 of the 71 nucleotides that constitute the *CYC1* promoter 5′UTR.

The promoter is the place on the DNA where transcription starts. Hence, it represents a fundamental component of synthetic gene circuits. A set of *S. cerevisiae* RNA polymerase II-dependent promoters are characterized by four main elements: The upstream activating sequence (UAS), the TATA box, the transcription start site (TSS), and the 5′UTR (untranslated region). Each of the first three elements can be present in multiple copies [6]. The core promoter is defined as the sequence composed of the TATA box(es), the TSS(s), and the 5′UTR (see Figure 1A). In addition, a considerable number of TATA-less promoters have been identified in the yeast genome [7].

The UASs in *S. cerevisiae*, such as the enhancers in higher eukaryotes, are bound by activator proteins that recruit the promoter RNA polymerase II together with the general transcription factor [8]. UASs are located ~100–1400 nucleotides upstream of the core promoter [6].

A consensus sequence for the yeast TATA box has been proposed by Basehoar et al. [9] as TATAWAWR (W: T or A; R: G or A). However, both the composition and length of the TATA box are not unambiguously defined [10]. In our analysis, we followed the work by Wobbe et al. [11] and considered the TATA box as a heptamer rich in thymine (T) and adenine (A) with the possible sporadic presence of a single cytosine (C) or guanine (G). The transcription initiation rate (i.e., the promoter strength) depends also on the actual TATA box sequence [12,13,14]. Moreover, the location and activity of the transcription start sites are constrained by the position of the TATA box(es) [15]. Indeed, a TATA box can activate only TSSs that are placed from 40 up to 120 nt downstream [16].

Constitutive endogenous promoters in *S. cerevisiae* play an important role into synthetic gene circuits since they guarantee a relatively stable protein (or RNA) expression level under different conditions [17]. However, not many native promoters have been characterized deeply, which implies a limited dynamic range for gene expression within synthetic circuits. To overcome this problem, libraries of synthetic promoters of different strengths have been developed using error-prone PCR on a single template [18] or building hybrid promoters by combining UASs and core sequences from different yeast promoters [19,20]. These synthetic promoters, however, are not orthogonal to *S. cerevisiae* cells and could also undergo homologous recombination with their original copy in the yeast genome.

To achieve orthogonality, either fully synthetic or foreign promoters shall be adopted. Viral promoters, for instance, are strongly active in mammalian cells and the cytomegalovirus promoter (pCMV—throughout this paper, the promoters’ names will generally start with a lower case “*p*”) has been reported to be working in *S. cerevisiae,* as well [21,22,23,24]. In general, though, the pathways that activate viral promoters in higher eukaryotes are not conserved in yeast, making those promoters unfunctional. Moreover, the distance between the TATA box and the TSS—in the virus but also in most eukaryotes—is far below the 40 nt required in *S. cerevisiae* for transcription activation [25].

Taking inspiration from the paper by Ede et al. [25], we constructed, in this work, a new library of *S. cerevisiae* constitutive promoters based on foreign core promoters—mainly from viruses plus one from human cells and another from the yeast *Schizosaccharomyces pombe*. To make them functional in *S. cerevisiae*, we extended them with an 87 nt-long sequence, termed pCYC1noTATA [26], that contains the full 5′UTR of the yeast *CYC1* promoter where at least six TSSs are present (see Figure 1B). The pCYC1noTATA provided the necessary spacing for the TATA box of the foreign core promoters to activate transcription from one or more TSSs belonging to pCYC1.

After testing the working of the new synthetic core promoters, we focused on the medium-strength promoter obtained from pSV40 (the simian virus 40) and studied how to modulate its strength by: (1) Using different TATA boxes obtained by mutating, in each position, the strong TATAAAA sequence; (2) varying the distance between a selected, moderately strong, TATA box (TATATAT) and the TSS; (3) removing nucleosomes from the promoter sequence; (4) placing UASs (of variable length) upstream of the core promoter; (5) varying the distance between the UASs and the TATA box. The best results from each of these analyses were used together to improve the transcription initiation rate of both the strongest and the weakest core promoter in our initial library.

In addition to building, overall, 59 new yeast synthetic promoters that shared a minimal homologous region with the *S. cerevisiae* genome, we found out important rules for designing and placing the sequences of both TATA boxes and UASs in order to modulate gene expression in yeast. Thus, our promoters and, more in general, our results can be used to fine-tune the working of synthetic transcriptional networks and metabolic pathways in *S. cerevisiae*.

## 2. Results and Discussion

### 2.1. Constructing New Synthetic Core Promoters

**Figure 2:**
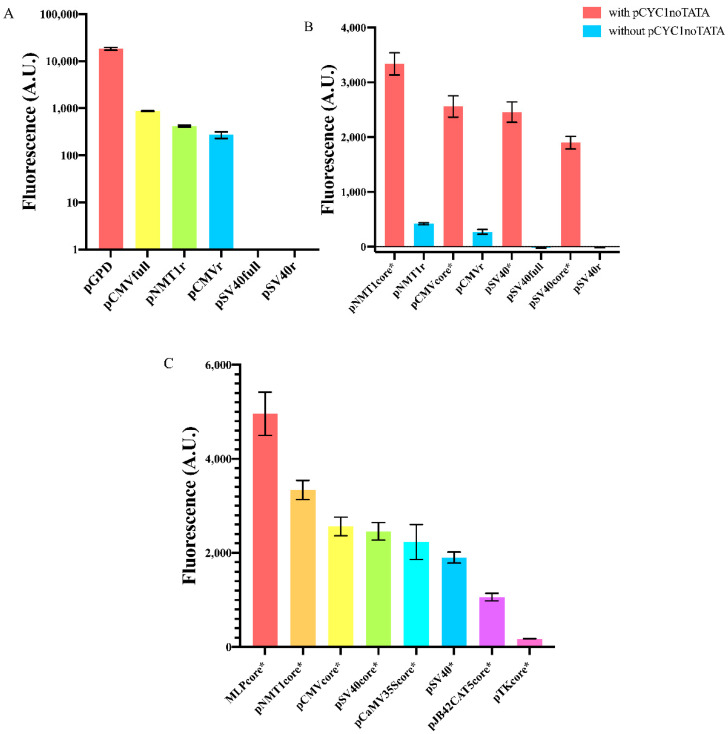
Fluorescence expression from foreign promoters and yeast synthetic core promoters. (**A**) Full or reduced foreign promoter sequences appear either unfunctional (pSV40full, pSV40r) or extremely weak if compared to the strong yeast constitutive *GPD* promoter. Fluorescence intensity, on the y-axis, is represented in a logarithmic scale and expressed in arbitrary units (AU). (**B**) Synthetic core promoters constructed by extending foreign promoters with pCYC1noTATA show a dramatic enhancement in fluorescence expression. (**C**) Comparison of the mean fluorescence expressed by our eight new synthetic core promoters. Each mean fluorescence value comes from three independent experiments.

We started our work by measuring the fluorescence level expressed, in *S. cerevisiae*, by two viral promoters commonly used in mammalian cells—pCMV [27,28] and pSV40 [29]—and a variant of the strong *S. pombe NMT1* promoter (no message in thiamine) [30]. As for the viral promoters, we considered both the full (pCMVfull and pSV40full) and a reduced sequence obtained by removing nucleotides upstream of the TATA box (pCMVr and pSV40r). As for pNMT1, we analyzed a reduced sequence only (pNMT1r). The nucleotides we removed from the original promoter sequences contain, mostly, binding sites for transcription factors that are not expressed in *S. cerevisiae* (e.g., those responding to thiamine in *S. pombe*) and, therefore, represent a potential genomic burden that might have a negative effect on the promoter transcription efficiency. Both versions of pSV40 were unfunctional, whereas pCMVfull and the reduced CMV and *NMT1* promoters turned out to be very weak, by expressing only 4.7, 1.5, and 2.3% of the fluorescence driven by the strong *GPD* promoter (see Figure 2A and Tables S1 and S2).

As mentioned in the Introduction, foreign promoters are usually not activated in *S. cerevisiae*. Core promoters retain only the elements strictly necessary for RNA polymerase II binding without the help of any activator proteins. Therefore, we reasoned that the main hurdle to gene expression by foreign core promoters was the short distance between the TATA box and the TSS. At least 40 nt between a TATA box and a TSS are considered to be necessary to allow transcription initiation. It should be noted, though, that Redden et al. [31] showed that minimal yeast synthetic promoters are operative with only 30 nt separating the TATA box from the TSS. In our first test, all the promoters we considered were characterized by even shorter distances between the TATA box and the TSS: 22 nt (pCMVfull/r) [21], 21 nt (pSV40full/r), and 20 nt (pNMT1r). As we have seen, only pSV40full/r were completely OFF, whereas pCMVfull/r and pNMT1r were very weak. We explain this discrepancy with the fact that pCMV and pNMT1 contain a strong TATA box (TATATAA, see below) apparently able to activate TSSs closer than 40 nt. In contrast, pSV40 hosts a very weak TATA signal (TATTTAT), clearly unable to recruit RNA polymerase II to the near TSSs.

We reckoned that we could enhance the performance of these foreign promoters by simply increasing the spacing between the TATA box and the TSS. To this aim, we first designed three new short core promoters (pCMVcore, pSV40core, and pNMT1core) and a longer one also derived from the viral SV40 promoter (we will refer to it simply as pSV40) based on little changes on the sequences in [25] (the viral promoters) and [32] (pNMT1core) that are known to work well either in mammalian or *S. pombe* cells. More precisely, we wanted to have (mainly) minimal core promoters starting with a TATA box. Hence, with the only exception of pCaMV35Score (see below), from the promoters published in [25] and [32] we kept only the sequences between the TATA box and the end of the 5′UTR. It should be noted that, along both pSV40 and pSV40core, we replaced the original TATA box, TATTTAT, with the stronger motif TATAAAA. Then, we extended the core promoters and pSV40 with the 87 nt long pCYC1noTATA that ensured to have over 40 nt between the TATA box and, at least, one TSS. Indeed, pCYC1noTATA contains 16 nt upstream of the TSS at position +1 and the whole 71 nt-long 5′UTR of the *CYC1* promoter, where six TSSs have been detected. In a previous work from our lab [26], we already proved that novel synthetic *S. cerevisiae* promoters can be successfully built by placing pCYC1noTATA downstream of a TATA box-like sequence (from natural or synthetic terminators). In the extended version of pCMVcore, pCMVcore* (from now on we will indicate with a “*” any promoter extended with pCYC1noTATA) the TATA box is able to activate all six TSSs since the TATA box-TSS distance varies from 51 to 93 nt. In contrast, pSV40core* would fail to start transcription from the TSS at position 35 and 43 (125 and 133 nt far from the TATA box, respectively), whereas pNMT1core* activates only the TSS at position +1 (116 nt distant from the TATA box). As shown in Figure 2B, pCMVcore* showed a 9.4-fold increase in fluorescence expression with respect to pCMVr and pNMT1core* manifested an 8.0-fold increment in fluorescence compared to the original (pNMT1r) sequence. Moreover, both pSV40* and pSV40core* became functional with a strength between the yeast *CYC1*core and *ACT1* promoter (see Table S1).

Therefore, we decided to build more synthetic promoters by extending, with pCYC1noTATA, the core sequence of other viral and eukaryotic promoters.

Following [25], we selected the core sequence of the three viral promoters: MLP (Major Late Promoter [33]), CaMV35S (cauliflower mosaic virus 35S) promoter [34], TK (Herpes simplex thymidine kinase) promoter [35], and the human pJB42CAT5 (associated with the *junB* gene [36]). Overall, we built eight new synthetic core promoters from yeast, mammalian, and viral origin. The strongest MLPcore* expressed a 28.3-fold higher fluorescence than the weakest pTKcore* (see Figure 2C). Interestingly, MLPcore* turned out to be 1.9-fold stronger than the constitutive *TEF1* promoter—widely employed in yeast synthetic gene circuits—whereas the pTKcore* strength was comparable with that of the weakest synthetic promoter previously built in our lab and termed “truncated_pCYC1”, where we just shortened pCYC1 5′UTR down to 24 nt [37] (details about the number of TSSs activated by each of these promoters and the distance between TSSs and the TATA box are presented in Table S3). 

Taken together, these results point out that both the distance between the TATA box and the TSSs and the number of activated TSSs can be used as parameters to change the promoter strength. Moreover, unfunctional foreign promoters can be turned into working ones just by extending them with a reasonably long sequence characterized by the presence of one or more TSSs.

### 2.2. Expanding the Promoter Library by Mutating the TATA Box

The actual sequence of the TATA box plays a non-negligible role in gene expression [12,13,14]. As the first confirmation to this assertion, we destroyed the original TATA box of MLPcore*, pCMVcore*, pSV40core*, and pSV40* by replacing it with a heptamer rich in guanine (GCGGGGG). This change caused a significant drop in fluorescence that, however, was not completely extinguished but reached a value close to that associated with pCYC1noTATA alone (see Table S1). We previously measured this fluorescence value to quantify the leakage effects due to the RNA polymerase II unspecific binding to the DNA [26]. The highest decrease, 14.2-fold, was registered on MLPcore* (see Figure S1).

We selected, then, a mid-strength synthetic core promoter, pSV40core*, in order to study how point mutations along the TATA box could modulate fluorescence expression. The original TATA box of pSV40 is TATTTAT. Initially, we replaced it with the sequence TATAAAA that, according to Wobbe et al. [11], is the second strongest TATA box in yeast, overcome only by TATAAAT. It should be noted, though, that the analysis by Wobbe et al. has two important differences with respect to ours: (1) The promoter they used as a template is the yeast native pTFIID; and (2) the quantitative results concerning all the TATA-box variants they took into account came from in vitro tests.

**Figure 3:**
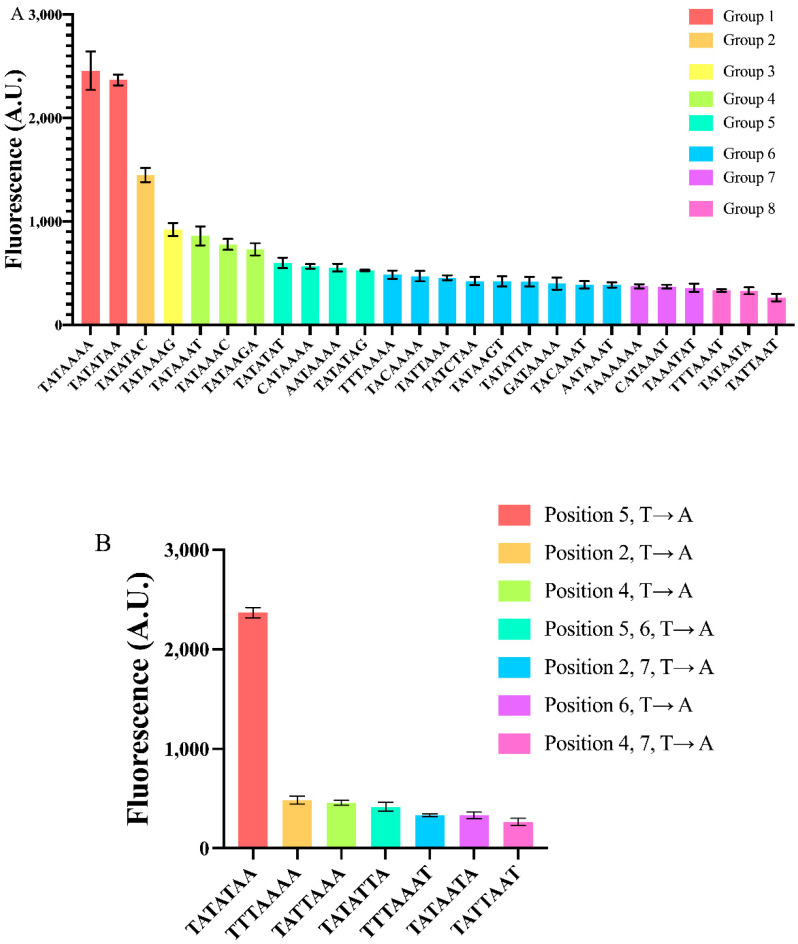
Constructing yeast synthetic core promoters via mutations on the TATA box. (**A**) Twenty-six core promoters have been realized via mutation(s) on the TATA box of pSV40core*(TATAAAA-90). They were distributed into eight groups depending on their strength. Groups were determined by both the two-sided Welch t–test and one-way ANOVA. (**B**) Effects of T → A mutation at different TATA box positions. Each mean fluorescence value comes from three independent experiments.

Overall, we constructed 25 variants of the pSV40core*(TATAAAA) via single or multiple mutations on the seven nucleotides belonging to the TATA box. The highest mean fluorescence value was detected from TATAAAA, the lowest (only 11% of the maximal one) from TATTAAT. By making a statistical analysis based on both one-way ANOVA and the two-sided Welch’s *t*-test (see “Material and Methods”) we divided our 26 core promoters into eight groups according to their fluorescence expression (see Figure 3A). We are aware of the fact that this classification has some limitations. For instance, the SV40core* promoters containing TATAAAG and TATAAAT are not significantly different, in statistical terms, under the two-sided Welch’s *t*-test (*p*-value = 0.98). However, only the TATAAAG-containing promoter is significantly different from the other two promoters in Group 4. Thus, we decided to assign pSVcore*(TATAAAG) to a different group (number 3), that contains no other instances. The two strongest TATA boxes (the above-cited TATAAAA together with TATATAA), which forms Group 1, gave an average fluorescence level corresponding to about 91% of that of the constitutive *ACT1* promoter. The only promoter in the second group, pSVcore*(TATATAC), is roughly as strong as the core *CYC1* promoter [16] that retains the three TATA boxes of the full pCYC1 [16]. From the third to the eighth group, the promoters are rather weak (see Tables S4–S6). The strongest of them almost matches a different synthetic promoter constructed in our lab (termed genCYC1t_pCYC1noTATA [26]) that we recently used to study the activity of type V anti-CRISPR proteins in *S. cerevisiae* [37].

From our results, we can make interesting considerations about the effects of diverse mutations on different positions along the TATA box.

As for single point mutations on TATAAAA, the least detrimental one was the change of an adenine to a thymine (T → A) at position 5, which did not significantly alter the core promoter strength.

We checked all the possible three mutations at position 7. Each of them induced a more consistent decrease in fluorescence expression, the highest (68%) was due to the C → A substitution. Interestingly, this result is consistent with [11]. In contrast, the outcome of the other two mutations are quite different from the values reported in [11]: G → A provoked a 62.5% fluorescence reduction rather than 23%; even more surprisingly, T → A resulted in a 65% drop in fluorescence in our experiments, whereas Wobbe et al. detected a 7% increase. It should be noted that during the construction of pJB42CATcore*, a G → A mutation happened at position 7 of the TATA box, which drastically lowered (51%) the expression level of this core promoter, as well (see Figure S2).

We made each of the possible three point mutations also at position 1. The decreases in fluorescence were significantly higher with respect to those concerning position 7. In particular, the G → T mutation at position 1 reduced fluorescence of even 84%. Single mutations at position 2, 3, and 4 affected fluorescence in a similar way to those at position 1 (see Table S6). Mutations at position 6 were the least homogeneous since G → A caused a 30% fluorescence reduction, whereas T → A suppressed fluorescence up to 87%.

Double and triple simultaneous mutations along TATAAAA confirmed the trend of reducing promoter strength with a fluorescence decrease ranging from 76 to 86% (see Table S6). The only remarkable exception was the double mutation T → A and C → A at positions 5 and 7, respectively. The promoter carrying the TATATAC sequence expressed 59% of the original pSVcore*(TATAAAA). Interestingly, TATATAC can be also regarded as a single point mutation (at position 7) on the other strong TATA box in Group 1. The decrease in fluorescence expression with respect to TATATAA is roughly 39%. By changing the final adenine with a thymine or a guanine, the drop in fluorescence level was more consistent: 75 and 78%, respectively. Therefore, the two strongest TATA boxes in our analysis do not share the same sensitivity to nucleotide change in the last position (see Figure S3).

Looking more in detail at the different kinds of point mutations, we saw that T → A is responsible for a strong decrease in fluorescence expression, unless it takes place (alone) at position 5. In particular, a simultaneous T → A mutation at both positions 5 and 6 returned an 83% fluorescence repression (see Figure 3B), pointing out how a thymine at position 6 can reduce promoter strength considerably.

In general, a TATA box mainly contains adenine and thymine but a single cytosine or guanine can be found in natural TATA boxes. The insertion of a single “C” or “G” into TATAAAA caused a reduction in fluorescence, though without following a precise pattern. At position 1, C → T is more favorable than G → T (and even A → T), whereas at position 7, G → A is more tolerated than C → A. In contrast, C → A has a relatively low effect if carried out at position 7 of TATATAA, whereas G → A and T → A have an apparent negative influence on gene expression (see Table S6).

Overall, we have seen how simple modifications on the TATA box of a synthetic core promoter span a 9.3-fold dynamic range in gene expression. In our analysis, TATAAAA and TATATAA resulted in being strong signals, whereas the weakest was TATTAAT that, interestingly, does not contain any cytosine, guanine or thymine at position 6—a feature that seemed to attenuate considerably DNA transcription. Moreover, differently from what was present in the literature, we found out that a thymine and a guanine at the seventh position cause a strong decrease in gene synthesis. This might indicate that context effects due the nucleotides directly upstream and downstream of the TATA box play a so far not understood role in DNA transcription, which needs further study.

### 2.3. Finding the Optimal Distance between the TATA Box and the TSS 

After ranking the TATA box heptamer according to the fluorescence they drove from pSV40core*, we looked for the optimal distance between the TATA box and the TSS to maximize gene expression.

A TATA box is believed to activate only TSSs that are placed from 40 up to 120 nt downstream of it [15]. However, as mentioned above, Redden et al. [31] managed to build working synthetic minimal promoters where the TATA box-TSS distance was reduced to 30 nt. Moreover, pCMVr and pNMT1r can drive mRNA synthesis, though at a low rate, despite the fact that their TSSs are just about 20 nt downstream of the TATA box. 

For this new analysis, we selected the configuration of pSV40* that contained the moderately weak TATATAT box (roughly 25% as strong as TATAAAA).

**Figure 4:**
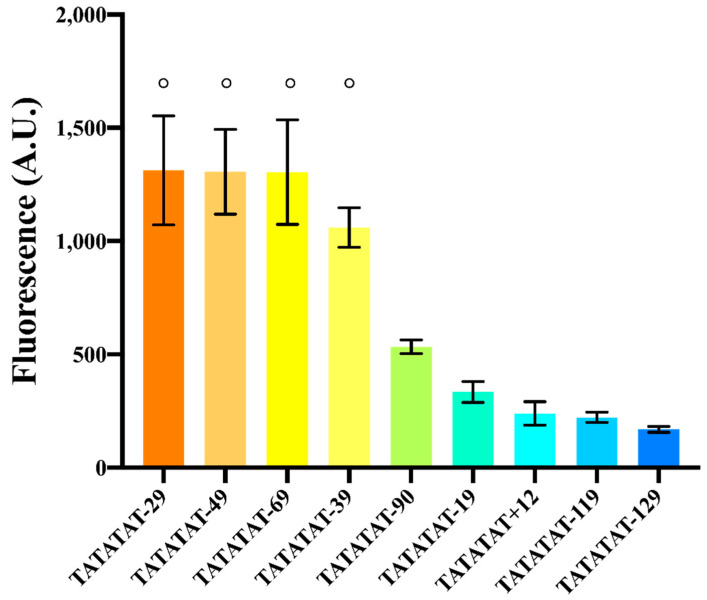
Effects on fluorescence expression due to the distance between the TATA box and the TSS. Experiments were carried out (in three different days) on variants of pSV40*(TATATAT). The symbol ° on top of different columns indicates that there is no significant statistical difference among the fluorescence levels of the corresponding promoters (*p*-value = 0.3704, one-way ANOVA).

We constructed nine variants of pSV40*(TATATAT) by varying the distance between the TATA box and TSS from −129 to +12 nt, being −90 nt the original one (a positive distance means that the TATA box was placed in the 5′UTR, downstream of the TSS at position +1). As shown in Figure 4 and Table S7, all promoters were functional, though with different strengths. This first observation underlines the fact that the 40-to-120 nt distance to activate transcription is not a strict rule. The TATA box starting 14 nt downstream of the TSS was probably able to activate another TSS located at position +43 [16].

Too short or too long distances (+12, −19, −119, and −129) determined low fluorescence expression, which confirmed the initial conjecture about the inoperativeness of foreign promoters in *S. cerevisiae* due to a non-optimal spacing between the TSS and TATA box. In contrast, promoters where TATATAT was −29, −39, −49 or −69 nt far from the TSS showed up to a ~2.5-fold increase in the fluorescence level of the original pSV40* (where TATATAT is 90 nt upstream of the TSS). The variant whose distance was −39 nt showed a lower mean fluorescence level. However, it was not significantly different in statistical terms from the other three. On the whole, our analysis shows that the region where the TATA box should lie in order to achieve maximal transcription goes roughly from position −30 to −70 with respect to the TSS.

### 2.4. Nucleosome Removal

As a further attempt to improve the strength of our new synthetic promoters, we tried to remove possible nucleosomes from their sequences [38,39]. In this task, we selected the pSV40* promoter with the moderately strong TATATAT box located 49 nucleotides upstream of the TSS. By following the procedure in [40], we run the NuPoP algorithm [41] to identify possible regions occupied by nucleosomes (usually reach in guanine and cytosine) and modify them with the addition of thymines, which disfavors nucleosome formation. Following our simulations, we had to insert three islands of 15 thymines each, within pSV40*(TATATAT), starting 170, 114, and 78 nt upstream of the TSS. The new promoter, termed pSV40*–45T, did not show, however, a significant improvement with respect to its original template (see Figure S4). We suppose that the modifications in the native promoter sequence altered somehow the normal RNA polymerase II binding nullifying, in this way, the potential benefit due to nucleosome removal.

### 2.5. Building New Synthetic Promoters Via the Addition of an UAS

After finalizing the construction and the investigation of our core promoters, we built on them novel hybrid promoters by placing an UAS upstream of their TATA box. UASs recruit activators of different strengths and, therefore, can increase considerably the transcription initiation rate of a promoter. Moreover, in *S. cerevisiae*, UASs should lie at a proper distance from the TATA box, i.e., between 100 and 1400 nt [6]. For this reason, long sequences encompassing both the activator site(s) and the “spacer” to the TATA (UAS-plus-spacer) were mainly utilized, so far, in the assembly of hybrid promoters in yeast [20].

Initially, we explored the effect of different UASs on DNA transcription from pSV40*(TATATAT−49) again. We chose the long UAS-plus-spacer sequence UAS_GPD(long)_ (532 nt) [42] and UAS_TEF1(long)_ (203 nt) [20,43]. In addition, we considered actual, short, UASs—neglected in previous yeast Synthetic Biology works—namely: The strong, bipartite, 40 nt long sequence from pGPD—UAS_GPD(40nt)_ [42]; the 20 nt-long RAP1 binding site along pTEF1—UAS_TEF1(RAP1bs)_ [44]; and the 59-long tripartite RAP1 site on pTEF2—UAS_TEF2(59nt)_ [45]. Finally, we also employed the strong, 30 nt-long, synthetic tripartite UAS_FEC_ built by Redden et al. [31]. All these three short UASs were placed 150 nt upstream of the TATA box.

**Figure 5:**
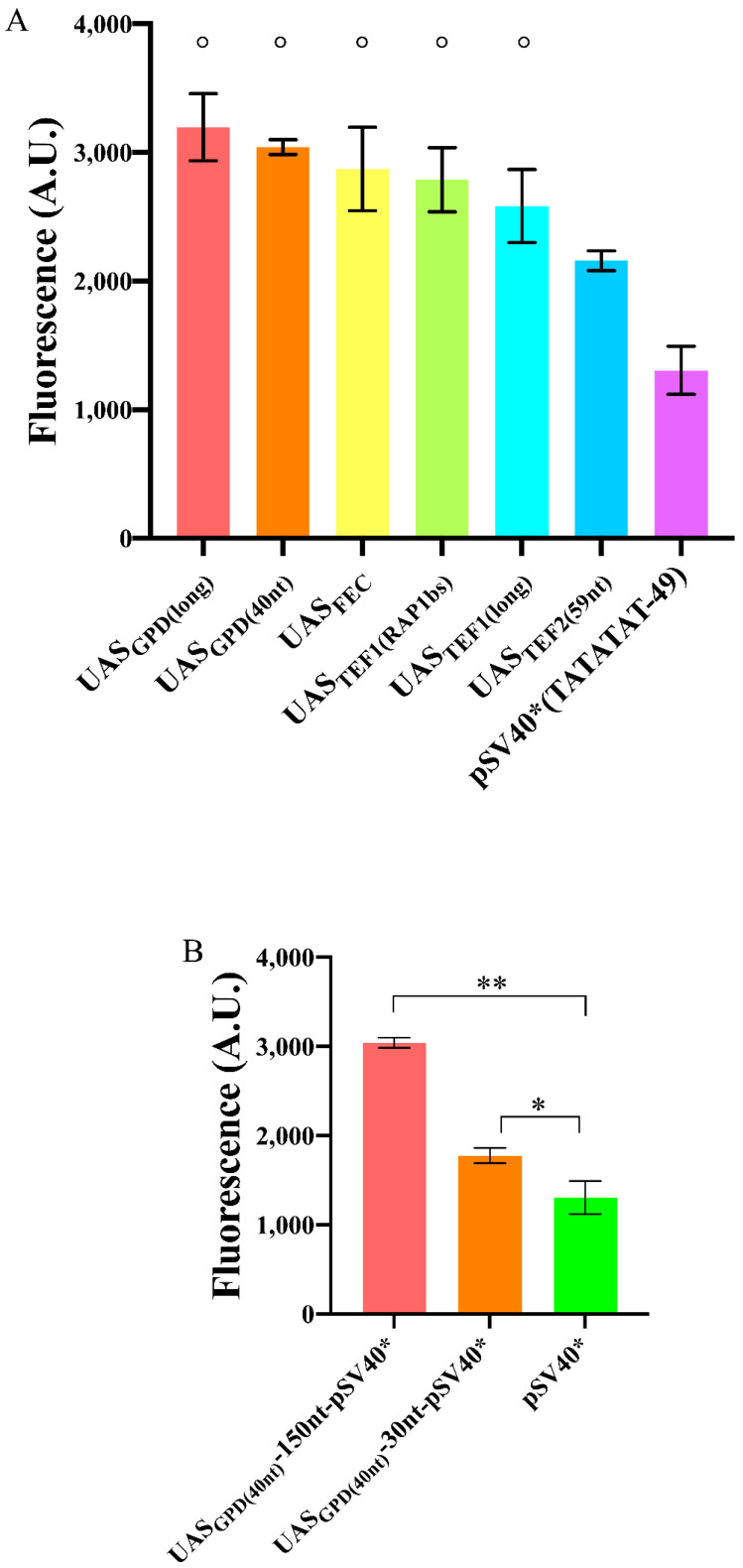
Enhancement in the fluorescence level of pSV40*(TATATAT-49) due to the presence of an UAS. (**A**) Most of the UASs considered in this work had a similar effect when placed 150 nt upstream of pSV40*(TATATAT-49). The symbol ° on top of five columns indicates no significant statistical difference (*p*-value = 0.1005, one-way ANOVA) among the corresponding fluorescence levels. (**B**) Variation in fluorescence expression due to different spacers between UAS_GPD(40nt)_ and pSV40*(TATATAT-49). The configuration with a 150 nt-long spacer was the most effective though 30 nt were enough to have a statistically significant increase in the fluorescence level of pSV40*(TATATAT-49). Each mean fluorescence value is the result of three independent experiments; * corresponds to a *p*-value < 0.05; ** to a *p*-value < 0.01 (two-sided Welch’s *t*-test).

Each UAS contributed to an increase in the fluorescence expressed by pSV40*(TATATAT-49). The least performant one was UAS_TEF2(59nt)_ (1.65-fold increase), the best one UAS_GPD(long)_ (2.45-fold increase). However, in statistical terms, UAS_GPD(long)_, UAS_GPD(40nt)_, UAS_TEF1(long)_, UAS_TEF1(RAP1bs)_, and UAS_FEC_ did not show any significant difference, pointing out that the length of synthetic promoters can be shortened remarkably by considering only endogenous activator binding sites as UASs (see Figure 5A and Table S8).

We also placed UAS_GPD(40nt)_ 150 nt upstream of the pSV40*-45t, which resulted in a 1.91-fold increase in the promoter strength (see Figure S4).

Taken together, our results showed that natural “minimal” UASs are capable of increasing, considerably, the promoter transcription rate when placed reasonably close (150 nt) to the TATA box.

Redden et al. [31], however, demonstrated that a much shorter spacer (30 nt) between UAS_FEC_ and a core promoter was enough to achieve a very high fluorescence level. This short distance permitted, as well, obtaining minimal synthetic promoters. We placed, therefore, UAS_GPD(40nt)_ just 30 nt upstream of the TATA box of pSV40*(TATATAT-49). Compared to the bare pSV40*(TATATAT-49), the increase in fluorescence was modest (1.36-fold), whereas a 150 nt spacer guaranteed a 2.33-fold enhancement. Therefore, a too-short distance between UAS and TATA box cannot be regarded as a universally valid strategy to enhance the promoter strength strongly (see Figure 5B).

### 2.6. Final Promoter Optimization

In the previous analyses, we found that the activity of SV40*-based promoters can be improved using the heptamer TATAAAA (or TATATAA) as a TATA box, setting the distance between the TATA box and the TSS to 49 nt, and placing the short UAS_GPD(40nt)_ 150 nt upstream of the TATA box.

According to our initial measurements, MLPcore* and pTKcore* were the strongest and the weakest synthetic core promoters obtained by extending their native sequences with pCYC1noTATA. In order to see if our findings had general validity, we modified both MLPcore* and pTKcore*, where necessary, with the same changes that were effective on pSV40*. Moreover, we checked if a reduction, on both promoters, of the distance between UAS_GPD(40nt)_ and the TATA box to 30 nt could give a considerable enhancement in gene expression.

**Figure 6:**
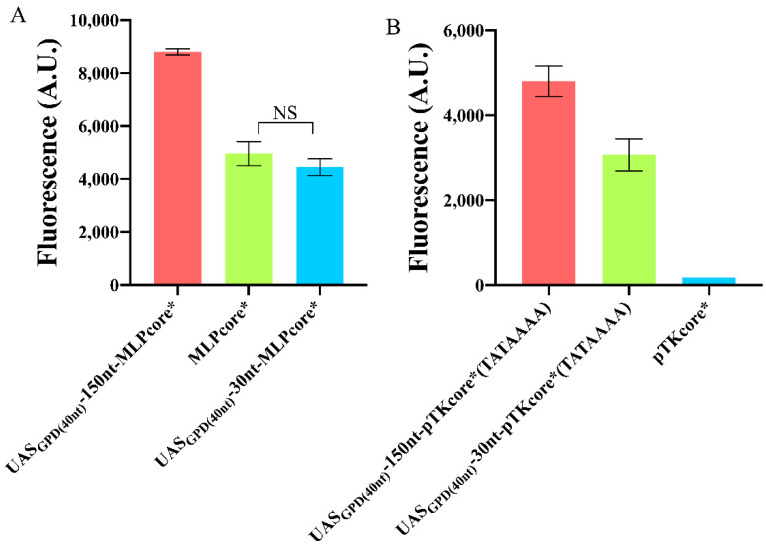
Variation in the fluorescence level of foreign promoters upon addition of an UAS. (**A**) A 150 nt spacer between UAS_GPD(40nt)_ and MLPcore* drastically improved fluorescence expression, whereas a 30 nt spacer had no significant effects (NS: *p*-value = 0.1961, two-sided Welch’s *t*-test). (**B**) The weak pTKcore*, modified with the strong TATA box—TATAAAA, underwent dramatic enhancement in fluorescence expression by placing UAS_GPD(40nt)_ both 30 and 150 nt upstream of the TATA box. Each mean fluorescence value comes from three independent experiments, NS: No significant statistical difference.

As for MLPcore*, its native TATA box coincides with TATAAAA and lies 43 nt upstream of the TSS, therefore in the optimal region for transcription (from −30 to −70). Thus, we constructed two variants of this promoter simply by adding UAS_GPD(40nt)_ either 30 or 150 nt upstream of the TATA box. In the former case, no significant difference was detected with respect to the original MLPcore*, whereas the latter provided a 1.77-fold increase in fluorescence expression. Moreover, the fluorescence intensity expressed by UAS_GPD(40nt)_−150nt-MLPcore* exceeded that of the strong yeast constitutive *TEF2* promoter (see Figure 6A and Table S1) such that this synthetic promoter was the strongest we managed to build in this work.

The TATA box of pTKcore* did not need a change in position either since it is located 67 nt upstream of the TSS. However, its composition (ATATTAA) is very different from the optimal one and had to be replaced. In this case, both configurations with 30 and 150 nt distancing UAS_GPD(40nt)_ from the TATA box induced a huge improvement in the promoter performance: 17.54- and even 27.45-fold, respectively (see Figure 6B). Therefore, we can conclude that, a spacer longer than 100 nt between a short, strong UAS and a strong TATA box represents the most reliable configuration, so far explored, for increasing the strength of synthetic promoters.

## 3. Conclusions

In this work, we constructed a large library of overall 59 new *S. cerevisiae* synthetic promoters based on foreign template sequences coming from viral and eukaryotic cells.

Foreign promoters, in yeast, are either unfunctional (such as pSV40) or poorly performant (e.g., pCMV) due to the absence of the activators necessary to recruit RNA polymerase II and the short distance between the TATA box and the TSS.

We managed to make foreign promoters work in *S. cerevisiae* by extending them with an 87 nt long sequence that included the whole yeast *CYC1* promoter 5′UTR, where at least six TSSs are present. This guaranteed a sufficient distance between the TATA box of the foreign promoter and some TSSs of *CYC1* promoter to initiate transcription. 

Our experiments permitted us to confirm—as already pointed out in [31]—that the general rule that claims that a TATA box can activate only TSS lying between 40 and 120 nt downstream shall not be considered as a real limit in the construction of yeast synthetic promoters. For instance, we showed that in our experiments the maximal promoter activity was obtained when the TATA box and the TSS were separated by, roughly, 30 up to 70 nucleotides. Importantly, the reciprocal position of the TATA box and the TSSs resents the scanning process of RNA polymerase II along part of the promoter sequence, which takes place during transcription initiation. A deeper understanding of this mechanism would further improve the design of new synthetic promoters in the yeast *S. cerevisiae* [46]. Moreover, we carefully indagated the effect of different TATA box sequences on transcription initiation. We confirmed that one of the strongest TATA boxes (here treated as heptamers) is TATAAAA even though not significantly more performant than TATATAA and, differently from what was published previously, much more efficient than TATAAAT [11]. By carrying out several point mutations, we realized how the substitution of an adenine with a thymine can lead to a remarkable decrease in the promoter strength. This is particularly evident when such a mutation happens at position 6, whereas position 5 seems the least sensitive to any change.

These initial experiments allowed the construction of 47 core promoters. We turned some of them into stronger synthetic promoters with the addition of an UAS. Importantly, we showed how short natural UASs from the constitutive *GPD*, *TEF1*, and *TEF2* promoters can highly enhance promoter activity, especially when placed 150 nt upstream of the TATA box. In contrast, a much lower distance (30 nt) does not appear to be always effective.

Overall, most of the core promoters in our library are relatively weak with three of them showing a moderate strength. The addition of a (short) UAS allowed the construction of stronger promoters, one of them overcoming the strong yeast constitutive *TEF2* promoter. Additionally, the usage of a foreign template lowers the risk of homologous recombination though both UAS and 5′UTR are taken from endogenous promoters and, therefore, prevent full orthogonality.

Developing synthetic leaders and UASs represent the next step to realize libraries of synthetic orthogonal promoters spanning a wider dynamic range in gene expression that will be beneficial for many diverse applications of Synthetic Biology.

## 4. Materials and Methods

### 4.1. Plasmids Construction

The backbone for all plasmids constructed in this work was the yeast integrative shuttle-vector pRSII406 (Addgene-35442, a gift from Steven Haase) [47]. The plasmids shown in Table S9, which contain the template for some of our synthetic promoters, were synthesized by Genewiz Inc., Suzhou (China).

Every transcription unit realized in this work is composed of a synthetic promoter followed by the HIS tag, the yeast enhanced green fluorescent protein (yEGFP) [48], and the *CYC1* terminator (CYC1t) [49]. All the DNA sequences used in this work are listed in the Supplementary Material.

Each plasmid containing a novel transcription unit was assembled via the Gibson assembly method [50]. To this aim, the pRSII406 vector was cut-open with Acc65I (NEB-R0599S) and SacI (NEB-R0156S). Touchdown PCR [51] was used to extract DNA sequences from their original plasmids. In this procedure, we employed Q5 Hot Start high-fidelity DNA polymerase (NEB-M0493S). PCR products were eluted from agarose gel by means of the AxiPrep DNA extraction kit (Axigen-AP-GX-250).

*E. coli* competent cells (strain DH5α, Life Technology,18263-012) were transformed with all the DNA fragments mixed in equimolar amount (30 s heat-shock at 42 °C) and grown overnight at 37 °C in LB (Luria-Bertani) plates (bacto-tryptone 10%, yeast extract 5%, NaCl 10%, agar 15%) supplied with ampicillin. Plasmid extraction from bacterial cells was carried out using standard methods [52]. All plasmids were sequenced via the Sanger method to check the correctness of the new synthetic constructs. A complete list of the plasmids realized in this work is given in Table S10.

### 4.2. Yeast Strain Construction

Each of our new plasmids was integrated into the genome of the yeast *S. cerevisiae* strain CEN.PK2-1C (MATa; his3D1; leu2-3_112; ura3-52; trp1-289; MAL2-8c; SUC2) kindly provided by Yuan Yingjin (Tianjin University, China). Plasmids (5 μg) were linearized with the restriction enzyme StuI (NEB-R0187V) that cuts inside the URA3 marker. Genomic integration was carried out according to the lithium-acetate protocol, as described in [53]. Transformed cells were grown on plates containing a synthetic selective medium lacking uracil (SD-URA; 2% glucose, 2% agar) for about 48 h at 30 °C. All yeast strains constructed in this work are listed in Table S11.

### 4.3. Flow Cytometry

Yeast cells were initially grown in a synthetic complete medium (SDC) at 30 °C, 240 RPM, for 18 h. Then, cells were 1:40 diluted. Green fluorescence was measured with a BD FACSVerse^TM^ Flow Cytometer (blue laser—488 nm, emission filter—527/32 nm). The FACS machine set-up was checked at the beginning and end of each experiment using fluorescent beads (BD FACSuite^TM^ CS&T Research Beads—650621). Measurements were considered as reliable only when the relative difference between the initial and final value of the peaks of the beads was not higher than 5%. Each strain was measured, at least, in three independent experiments (i.e., the cells were cultured on different days). During each experiment, 30,000 events were recorded.

### 4.4. Data Analysis

Data from the flow cytometer was analyzed with the flowcore R-Bioconductor package [54]. The mean background fluorescence, measured on the chassis strain (byMM584) that does not contain any fluorescence source, was subtracted from the mean fluorescence value associated with each engineered strain. A comparison between the fluorescence level of two strains was carried out via the two-sided Welch’s *t*-test. In contrast, one-way ANOVA was adopted to analyze groups of more than two strains (*p*-value < 0.05 was considered as significant to reject the null hypothesis).

## Data Availability

All the FCS files from FACS measurements have been deposited at FlowRepository (https://flowrepository.org/). They can be accessed through this link: http://flowrepository.org/id/RvFrORQgxxrkYh1hjZAjH2DgSu8x0beNWNCIWYVKUWWNAvUHWL0mxVruAaarRfBW.

## Supplementary Materials

The following are available online at https://www.mdpi.com/article/10.3390/ijms22115704/s1.
