# Supplementary material for: Novel S. cerevisiae Hybrid Synthetic Promoters Based on Foreign Core Promoter Sequences
Source: Int J Mol Sci. 2021 May 27;22(11):5704. doi: 10.3390/ijms22115704 (PMC8198421; doi:10.3390/ijms22115704)
Supplement: Supplementary file 1 [file ijms-22-05704-s001.zip › ijms-1214262-supplementary.pdf]

Novel *S. cerevisiae* hybrid synthetic promoters based on foreign core promoter sequences

<u>Supplementary Material</u>

Xiaofan Feng[1] and Mario Andrea Marchisio[1,*]
[1]School of Pharmaceutical Science and Technology, Tianjin University, 92 Weijin Road, 300072 Tianjin, China
*corresponding author

Table S1. Mean fluorescence expressed by constitutive natural and synthetic yeast promoters SD: standard deviation. Both mean fluorescence values and SD are calculated on three independent measurements.

Promoter	Average fluorescence (AU)	SD
pGPD	18390.48	1210.51
pTEF2	8063.26	56.68
pTEF1	2669.80	106.90
pACT1	2650.94	92.96
pCYC1core	1481.85	21.23
genCYC1t-pCYC1noTATA	937.02	54.86
pCYC1noTATA	343.64	25.07
truncated_pCYC1	179.19	17.79

Table S2. Mean fluorescence values from the foreign promoters considered in this work. Each value is the result of three independent experiments. SD: standard deviation.

Promoter	Average fluorescence (AU)	SD
pNMT1r	419.83	18.82
pCMVfull	872.15	14.52
pCMVr	272.1	43.61
pSV40full	-11.30	-
pSV40r	-18.30	-

Table S3. Mean fluorescence values expressed by foreign promoters extended with pCYC1noTATA. Each value refers to three independent experiments. SD: standard deviation.

Promoter	Average fluorescence (AU)	SD	Activated TSS	TATA box-TSS distance
MLPcore*	4959.87	459.23	6	44-86
pNMT1core*	3337.97	202.72	1	116
pCMVcore*	2560.68	196.42	6	51-93
pSV40core*	2457.5	185.9	4	91-115
pCAMV35Score*	2229.93	372.75	6	42-84
pSV40*	1900.63	116	4	91-115
pJB42CAT5core*	1062.99	82.03	4	91-115
pTKcore*	174.93	1.06	6	68-110

Table S4. Mean fluorescence expressed by variants of pSVcore* after mutating the strong TATA box, TATAAAA. Positions along the TATA box where mutations occurred are indicated in the second column (SD: standard deviation. Both mean fluorescence values and SD are calculated on three independent measurements).

Group	Mutated Positions	TATA box	Average fluorescence (AU)	SD
1	No mutation	TATAAAA	2457.50	185.90
	5	TATATAA	2368.09	52.14
2	5,7	TATATAC	1448.15	68.88
3	7	TATAAAG	922.30	63.29
4	7	TATAAAT	859.54	91.02
	7	TATAAAC	778.31	52.47
	6	TATAAGA	730.16	59.34
5	5,7	TATATAT	599.24	51.56
	1	CATAAAA	566.83	21.25
	1	AATAAAA	553.26	37.22
	5,7	TATATAG	526.55	7.56
6	2	TTTAAAA	485.53	40.62
	3	TACAAAA	470.83	49.78
	4	TATTAAA	455.76	23.99
	4,5	TATCTAA	423.41	32.95
	6,7	TATAAGT	420.09	40.35
	5,6	TATATTA	417.55	48.62
	1	GATAAAA	398.94	45.95
	3,7	TACAAAT	387.46	58.21
	1,7	AATAAAT	386.95	36.36
7	3	TAAAAAA	373.79	25.37
	1,7	CATAAAT	369.97	19.95
	3,5,7	TAAATAT	358.53	17.60
8	2,7	TTTAAAT	332.58	39.81
	6	TATAATA	329.91	13.58
	4,7	TATTAAT	264.89	33.20

Table S5. Average fluorescence expressed by each of the eight groups of synthetic SVcore* promoterd (see Table S4). SD, the standard deviation, is calculated for the groups that contain more than a single promoter.

Group	Average fluorescence (AU)	SD
1	2412.80	44.70
2	1448.15	-
3	922.30	-
4	789.34	53.39
5	561.47	26.18
6	427.39	33.79
7	367.43	6.48
8	309.13	31.30

Table S6. Effects on the mean fluorescence expressed by synthetic core promoters built on pSVcore* via single and multiple mutations on the TATA box (see Table S4).

Mutated position	TATA box	Average fluorescence (AU)	Relative fluorescence
No mutation	TATAAAA	2457.50	1.00
1	CATAAAA	566.83	0.23
1	AATAAAA	553.26	0.23
1	GATAAAA	398.94	0.16
2	TTTAAAA	485.53	0.20
3	TACAAAA	470.83	0.19
3	TAAAAAA	373.79	0.15
4	TATTAAA	455.76	0.19
5	TATATAA	2368.09	0.96
6	TATAAGA	730.16	0.30
6	TATAATA	329.91	0.13
7	TATAAAG	922.30	0.38
7	TATAAAT	859.54	0.35
7	TATAAAC	778.31	0.32
1,7	AATAAAT	386.95	0.16
1,7	CATAAAT	369.97	0.15
2,7	TTTAAAT	332.58	0.14
3,7	TACAAAT	387.46	0.16
4,7	TATTAAT	264.89	0.11
5,7	TATATAC	1448.15	0.59
5,7	TATATAT	599.24	0.24
5,7	TATATAG	526.55	0.21
6,7	TATAAGT	420.09	0.17
4,5	TATCTAA	423.41	0.17
5,6	TATATTA	417.55	0.17
3,5,7	TAAATAT	358.53	0.15

Table S7. Variation in the mean fluorescence expressed by pSV40* due to different position of the TATA box (TATATAT) with respect to the TSS. Each fluorescence level is the result of three independent experiments. SD indicates the standard deviation.

Promoter with distance between TATA box and TSS	Average fluorescence (AU)	SD
pSV40*(TATATAT-29)	1312.24	240.42
pSV40*(TATATAT-49)	1305.79	187.30
pSV40*(TATATAT-69)	1304.11	230.35
pSV40*(TATATAT-39)	1059.74	87.52
pSV40*(TATATAT-90)	533.86	30.68
pSV40*(TATATAT-19)	334.09	45.93
pSV40*(TATATAT+12)	239.47	51.45
pSV40*(TATATAT-119)	222.19	22.37
pSV40*(TATATAT-129)	169.17	13.14

Table S8. Effect on fluorescence expression due to different UASs placed 150 nt upstream of the TATA box of pSV40*(TATATA-49). The strong bipartite UAS from the *GPD* promoter induced the highest increase in fluorescence level. Mean fluorescence and standard deviation (SD) are calculated on three independent measurements.

Name of promoters	Average fluorescence (AU)	SD
$UAS_{GPD(long)}$	3197.30	260.84
$UAS_{GPD(40nt)}$	3041.61	57.46
$UAS_{TEF1(long)}$	2583.32	284.11
$UAS_{(FEC)}$	2871.84	325.33
$UAS_{TEF1(RAP1bs)}$	2787.17	250.13
$UAS_{TEF2(59nt)}$	2155.80	77.75
pSV40(TATATAT-49)	1305.79	187.30

Table S9. Plasmids synthesized, in this work, by Genewiz Inc. Suzhou (China).

Plasmid name	Construct
pMM861	pUC57-pSV40*(TATAAAA-90)
pMM901	pUC57-pSV40*(GCGGGGG-90)
pMM999	pUC57-CaMV35Score*
pMM1000	pUC57-pTKcore*
pMM1025	pUC57-pSV40*(TATATAT-39)
pMM1026	pUC57-pSV40*(TATATAT-90)
pMM1027	pUC57-pSV40*(TATATAT-129)
pMM1055	pUC57-pSV40*(TATATAT+12)
pMM1056	pUC57-pSV40*(TATATAT-19)
pMM1057	pUC57-pSV40*(TATATAT-119)
pMM1093	pUC57-pSV40*(TATATAT-29)
pMM1094	pUC57-pSV40*(TATATAT-49)
pMM1095	pUC57-pSV40*(TATATAT-69)
pMM1125	pUC57-pSV40*-45T
pMM1147	pUC57-$UAS_{GPD(40nt)}$-150nt-pSV40*(TATAAAA-49)
pMM1152	pUC57-$UAS_{GPD(40nt)}$-150nt-pSV40*(TATAAAA-49)-45T

Table S10. Plasmids assembled in this work.

Plasmid name	Construct
pMM863	pRSII406-pSV40*(TATAAAA)-ATG-HIStag-yEGFP-CYC1t
pMM902	pRSII406-pSV40*(GCGGGGG)-ATG-HIStag-yEGFP-CYC1t
pMM905	pRSII406-pSV40core*(TATAAAA)-ATG-HIStag-yEGFP-CYC1t
pMM926	pRSII406-pMLPcore*-ATG-HIStag-yEGFP-CYC1t
pMM931	pRSII406- pCMVcore*-ATG-HIStag-yEGFP-CYC1t
pMM944	pRSII406- pNMT1core*-ATG-HIStag-yEGFP-CYC1t
pMM946	pRSII406- pJB42CAT5core*-ATG-HIStag-yEGFP-CYC1t
pMM951	pRSII406-pSV40core*(GCGGGGG)-ATG-HIStag-yEGFP-CYC1t
pMM957	pRSII406-pSV40core*(AATAAAA)-ATG-HIStag-yEGFP-CYC1t
pMM958	pRSII406-pCMVcore*(GCGGGGG)-ATG-HIStag-yEGFP-CYC1t
pMM981	pRSII406-pSV40core*(TAAAAAA)-ATG-HIStag-yEGFP-CYC1t
pMM982	pRSII406-pSV40core*(TATAAAT)-ATG-HIStag-yEGFP-CYC1t
pMM983	pRSII406-pSV40 core*(TATATAA)-ATG-HIStag-yEGFP-CYC1t
pMM984	pRSII406-pSV40core*(TATATAT)-ATG-HIStag-yEGFP-CYC1t
pMM985	pRSII406-MLPcore*(GCGGGGG) -ATG-HIStag-yEGFP-CYC1t
pMM991	pRSII406-pSV40core*(CATAAAA)-ATG-HIStag-yEGFP-CYC1t
pMM992	pRSII406-pSV40core*(TACAAAT)-ATG-HIStag-yEGFP-CYC1t
pMM993	pRSII406-pSV40core*(TATAAGA)-ATG-HIStag-yEGFP-CYC1t
pMM1002	pRSII406-pSV40core*(TATAAGT)-ATG-HIStag-yEGFP-CYC1t
pMM1003	pRSII406-pSV40core*(TACAAAA)-ATG-HIStag-yEGFP-CYC1t
pMM1007	pRSII406-pSV40core*(TAAATAT)-ATG-HIStag-yEGFP-CYC1t
pMM1010	pRSII406-pCaMV35Score*-ATG-HIStag-yEGFP-CYC1t
pMM1011	pRSII406-pTKcore*-ATG-HIStag-yEGFP-CYC1t
pMM1012	pRSII406-pSV40core*(AATAAAT)-ATG-HIStag-yEGFP-CYC1t
pMM1013	pRSII406-pSV40core*(TTTAAAT)-ATG-HIStag-yEGFP-CYC1t
pMM1014	pRSII406-pSV40core*(TATTAAA)-ATG-HIStag-yEGFP-CYC1t
pMM1015	pRSII406-pSV40core*(TATTAAT)-ATG-HIStag-yEGFP-CYC1t
pMM1016	pRSII406-pSV40core*(CATAAAT)-ATG-HIStag-yEGFP-CYC1t
pMM1028	pRSII406-pSV40*(TATATAT-39)-ATG-HIStag-yEGFP-CYC1t
pMM1029	pRSII406-pSV40*(TATATAT-90)-ATG-HIStag-yEGFP-CYC1t
pMM1030	pRSII406-pSV40*(TATATAT-129)-ATG-HIStag-yEGFP-CYC1t
pMM1033	pRSII406-pSV40core*(TTTAAAA)-ATG-HIStag-yEGFP-CYC1t
pMM1047	pRSII406-pSV40core*(TATAAAC)-ATG-HIStag-yEGFP-CYC1t
pMM1048	pRSII406-pSV40core*(TATAAAG)-ATG-HIStag-yEGFP-CYC1t
pMM1049	pRSII406-pSV40core*(TATATAC)-ATG-HIStag-yEGFP-CYC1t
pMM1050	pRSII406-pSV40core*(TATATAG)-ATG-HIStag-yEGFP-CYC1t
pMM1051	pRSII406-pSV40core*(TATAATA)-ATG-HIStag-yEGFP-CYC1t
pMM1052	pRSII406-pSV40core*(TATCTAA)-ATG-HIStag-yEGFP-CYC1t
pMM1053	pRSII406-pSV40core*(TATATTA)-ATG-HIStag-yEGFP-CYC1t
pMM1054	pRSII406-pSV40core*(GATAAAA)-ATG-HIStag-yEGFP-CYC1t

pMM1058	pRSII406-pSV40*(TATATAT+12)-ATG-HIStag-yEGFP-CYC1t
pMM1059	pRSII406-pSV40*(TATATAT-19)-ATG-HIStag-yEGFP-CYC1t
pMM1060	pRSII406-pSV40*(TATATAT-119)-ATG-HIStag-yEGFP-CYC1t
pMM1097	pRSII406-pSV40*(TATATAT-29)-ATG-HIStag-yEGFP-CYC1t
pMM1098	pRSII406-pSV40*(TATATAT-49)-ATG-HIStag-yEGFP-CYC1t
pMM1099	pRSII406-pSV40*(TATATAT-69)-ATG-HIStag-yEGFP-CYC1t
pMM1106	pRSII406-$UAS_{GPD(long)}$-150nt-pSV40*(TATATAT-49)-ATG-HIStag-yEGFP-CYC1t
pMM1107	pRSII406-$UAS_{GPD(40nt)}$-150nt-pSV40*(TATATAT-49)-ATG-HIStag-yEGFP-CYC1t
pMM1108	pRSII406-$UAS_{TEF2(59nt)}$-150nt-pSV40*(TATATAT-49)-ATG-HIStag-yEGFP-CYC1t
pMM1126	pRSII406-pSV40*-45T-HIStag-yEGFP-CYC1t
pMM1127	pRSII406-UAS_{FEC}-150nt-pSV40*(TATATAT-49)-ATG-HIStag-yEGFP-CYC1t
pMM1128	pRSII406-$UAS_{TEF1(long)}$-150nt-pSV40*(TATATAT-49)-ATG-HIStag-yEGFP-CYC1t
pMM1129	pRSII406-$UAS_{TEF1(RAP1bs)}$-150nt-pSV40*(TATATAT-49)-ATG-HIStag-yEGFP-CYC1t
pMM1161	pRSII406-$UAS_{GPD(40nt)}$-30nt-MLPcore*-ATG-HIStag-yEGFP-CYC1t
pMM1162	pRSII406-$UAS_{GPD(40nt)}$-30nt-pSV40*(TATATAT-49) -ATG-HIStag-yEGFP-CYC1t
pMM1163	pRSII406-$UAS_{GPD(40nt)}$-150nt-pSV40*-45T-ATG-HIStag-yEGFP-CYC1t
pMM1166	pRSII406-$UAS_{GPD(40nt)}$-30nt-pTKcore*(TATAAAA)-ATG-HIStag-yEGFP-CYC1t
pMM1183	pRSII406-$UAS_{GPD(40nt)}$-150nt-MLPcore*-ATG-HIStag-yEGFP-CYC1t
pMM1184	pRSII406-$UAS_{GPD(40nt)}$-150nt-pTKcore*(TATAAAA)-ATG-HIStag-yEGFP-CYC1t

Table S11. Yeast strains engineered in this work.

Strain name	Genotype
byMM783	byMM584 pMM863::URA3
byMM868	byMM584 pMM902::URA3
byMM869	byMM584 pMM905::URA3
byMM871	byMM584 pMM951::URA3
byMM872	byMM584 pMM926::URA3
byMM873	byMM584 pMM931::URA3
byMM874	byMM584 pMM944::URA3
byMM876	byMM584 pMM946::URA3
byMM893	byMM584 pMM957::URA3
byMM894	byMM584 pMM958::URA3
byMM900	byMM584 pMM985::URA3
byMM901	byMM584 pMM981::URA3
byMM902	byMM584 pMM982::URA3
byMM903	byMM584 pMM983::URA3
byMM904	byMM584 pMM984::URA3
byMM905	byMM584 pMM991::URA3
byMM906	byMM584 pMM992::URA3
byMM907	byMM584 pMM993::URA3
byMM916	byMM584 pMM1007::URA3
byMM918	byMM584 pMM1002::URA3
byMM919	byMM584 pMM1003:URA3
byMM920	byMM584 pMM1010::URA3
byMM921	byMM584 pMM1011::URA3
byMM940	byMM584 pMM1012::URA3
byMM941	byMM584 pMM1013::URA3
byMM942	byMM584 pMM1016::URA3
byMM945	byMM584 pMM1014::URA3
byMM947	byMM584 pMM1028::URA3
byMM948	byMM584 pMM1029::URA3
byMM949	byMM584 pMM1030::URA3
byMM970	byMM584 pMM1015::URA3
byMM971	byMM584 pMM1033::URA3
byMM972	byMM584 pMM1047::URA3
byMM974	byMM584 pMM1048::URA3
byMM975	byMM584 pMM1049::URA3
byMM976	byMM584 pMM1050::URA3
byMM977	byMM584 pMM1051::URA3
byMM978	byMM584 pMM1052::URA3
byMM979	byMM584 pMM1053::URA3
byMM980	byMM584 pMM1054::URA3

byMM981	byMM584 pMM1058::URA3
byMM982	byMM584 pMM1059::URA3
byMM983	byMM584 pMM1060::URA3
byMM1142	byMM584 pMM1097::URA3
byMM1143	byMM584 pMM1098::URA3
byMM1144	byMM584 pMM1099::URA3
byMM1170	byMM584 pMM1106::URA3
byMM1171	byMM584 pMM1107::URA3
byMM1172	byMM584 pMM1108::URA3
byMM1203	byMM584 pMM1127::URA3
byMM1204	byMM584 pMM1126:URA3
byMM1205	byMM584 pMM1129::URA3
byMM1206	byMM584 pMM1128::URA3
byMM1230	byMM584 pMM1161::URA3
byMM1231	byMM584 pMM1162::URA3
byMM1232	byMM584 pMM1163::URA3
byMM1254	byMM584 pMM1166::URA3
byMM1255	byMM584 pMM1184::URA3
byMM1256	byMM584 pMM1183::URA3

DNA sequences used in this work

<u>Sequences from previously engineered yeast strains. In bold, when available, TATA boxes and TSSs.</u>

pCMVfull (pMM467; byMM331—putative TSS)

AATTGCATGAAGAATCTGCTTAGGGTTAGGCGTTTTGCGCTGCTTCGCGAT
GTACGGGCCAGATATACGCGTTGACATTGATTATTGACTAGTTATTAATAG
TAATCAATTACGGGGTCATTAGTTCATAGCCCATATATGGAGTTCCGCGTT
ACATAACTTACGGTAAATGGCCCGCCTGGCTGACCGCCCAACGACCCCCG
CCCATTGACGTCAATAATGACGTATGTTCCCATAGTAACGCCAATAGGGA
CTTTCCATTGACGTCAATGGGTGGACTATTTACGGTAAACTGCCCACTTGG
CAGTACATCAAGTGTATCATATGCCAAGTACGCCCCCTATTGACGTCAAT
GACGGTAAATGGCCCGCCTGGCATTATGCCCAGTACATGACCTTATGGGA
CTTTCCTACTTGGCAGTACATCTACGTATTAGTCATCGCTATTACCATGGT
GATGCGGTTTTGGCAGTACATCAATGGGCGTGGATAGCGGTTTGACTCAC
GGGGATTTCCAAGTCTCCACCCCATTGACGTCAATGGGAGTTTGTTTTGGC
ACCAAAATCAACGGGACTTTCCAAAATGTCGTAACAACTCCGCCCCATTG
ACGCAAATGGGCGGTAGGCGTGTACGGTGGGAGGTC**TATATAA**GCAGAG
CTCTCTGGCTAACTAG**A**GAACCCACTGCTTACTGGCTTATCGAAATTAATA
CGACTCACTATAGGGAGACCCAAGCTGGCTAGTT

pCMVr (pMM331; byMM134)
GTAGGCGTGTACGGTGGGAGGTC**TATATAA**GCAGAGCTCGTTTAGTGAAC
CG**T**CAGATCGCCTGGAGACGCCATCCACGCTGTTTTGACCTCCATAATA

pSV40full (pMM291; byMM205)
GGAGCTGTGGAATGTGTGTCAGTTAGGGTGTGGAAAGTCCCCAGGCTCCC
CAGCAGGCAGAAGTATGCAAAGCATGCATCTCAATTAGTCAGCAACCAGG
TGTGGAAAGTCCCCAGGCTCCCCAGCAGGCAGAAGTATGCAAAGCATGCA
TCTCAATTAGTCAGCAACCATAGTCCCGCCCCTAACTCCGCCCATCCCGCC
CCTAACTCCGCCCAGTTCCGCCCATTCTCCGCCCCATGGCTGACTAATTTT
TTT**TATTTAT**GCAGAGGCCGAGGCCGCCTCT**G**CCTCTGAGCTATTCCAGA
AGTAGTGAGGAGGCTTTTTTGGAGGCCTAGGCTTTTGCAAAAAGCTC

pSV40r (pMM328; byMM131)
TGCATCTCAATTAGTCAGCAACCAGGTGTGGAAAGTCCCCAGGCTCCCCA
GCAGGCAGAAGTATGCAAAGCATGCATCTCAATTAGTCAGCAACCATAGT
CCCGCCCCTAACTCCGCCCATCCCGCCCCTAACTCCGCCCAGTTCCGCCCA
TTCTCCGCCCCATGGCTGACTAATTTTTTT**TATTTAT**GCAGAGGCCGAGGC
CGCCTCT**G**CCTCTGAGCTATTCCAGAAGTAGTGAGGAGGCTTTTTTGGAG
GCCTAGGCTTTTGCAAAAAGCTC

pNMT1r (pMM338; byMM143)
AAACATTGAAGA**TATATAA**AGGAAGAGGAATCCTGGCATA**A**TCATCAATT
GAATAAGTTGAATTAATTATTTCAATCTCATTCTCACTTTCTGACTTATAGT
CGCTTTGTTAAATTGGC

<u>Sequences used to assemble plasmids in this work.</u>

pCYC1noTATA
TTCTTTCCTTATACAT**t**AGGACCTTTGCAGCATAAATTACTATACTTCTATA
GACACA CAAACACAAATACACACACTAAATTAATA

ATG-HISTtag-yEGFP
ATGAGCCATCATCATCATCATCACAGCGGATCCTCTAAAGGTGAAGAATT
ATTCACTGGTGTTGTCCCAATTTTGGTTGAATTAGATGGTGATGTTAATGG
TCACAAATTTTCTGTCTCCGGTGAAGGTGAAGGTGATGCTACTTACGGTAA
ATTGACCTTAAAATTTATTTGTACTACTGGTAAATTGCCAGTTCCATGGCC
AACCTTAGTCACTACTTTCGGTTATGGTGTTCAATGTTTTGCGAGATACCC

AGATCATATGAAACAACATGACTTTTTCAAGTCTGCCATGCCAGAAGGTT
ATGTTCAAGAAAGAACTATTTTTTTCAAAGATGACGGTAACTACAAGACC
AGAGCTGAAGTCAAGTTTGAAGGTGATACCTTAGTTAATAGAATCGAATT
AAAAGGTATTGATTTTAAAGAAGATGGTAACATTTTAGGTCACAAATTGG
AATACAACTATAACTCTCACAATGTTTACATCATGGCTGACAAACAAAAG
AATGGTATCAAAGTTAACTTCAAAATTAGACACAACATTGAAGATGGTTC
TGTTCAATTAGCTGACCATTATCAACAAAATACTCCAATTGGTGATGGTCC
AGTCTTGTTACCAGACAACCATTACTTATCCACTCAATCTGCCTTATCCAA
AGATCCAAACGAAAAGAGAGACCACATGGTCTTGTTAGAATTTGTTACTG
CTGCTGGTATTACCCATGGTATGGATGAATTGTACAAATAA

CYC1t
CATGTAATTAGTTATGTCACGCTTACATTCACGCCCTCCCCCCACATCCGC
TCTAACCGAAAAGGAAGGAGTTAGACAACCTGAAGTCTAGGTCCCTATTT
ATTTTTTTATAGTTATGTTAGTATTAAGAACGTTATTTATATTTCAAATTTT
TCTTTTTTTTCTGTACAGACGCGTGTACGCATGTAACATTATACTGAAAAC
CTTGCTTGAGAAGGTTTTGGGACGCTCGAAGGCTTTAATTTGCAAGCTATC

pSV40 (original template)
TGCATCTCAATTAGTCAGCAACCATAGTCCCGCCCCTAACTCCGCCCATCC
CGCCCCTAACTCCGCCCAGTTCCGCCCATTCTCCGCCCCATCGCTGACTAA
TTTTTTTT**TATTTAT**GCAGAGGCCGAGGCCGCCTC**G**GCCTCTGAGCTATTCC
AGAAGTAGTGAGCAGGCTTTTTTGGCGACCTAGGCTT

pSV40core (original template)
TATTTATGCAGAGGCCGAGGCCGCCTC**G**GCCTCTGAGCTATTCCAGAAGT
AGTGAGCAGGCTTTTTTGGCGACCTAGGCTT

pSV40-45T
GCATCTCAATTAGTCAGCAACCATAGTCCCGCCCCTAACTCCGCCCATCCC
GCCCCTAACTCCGCCCAGTTCCGCCCTTTTTTTTTTTTTTATTCTCCGCCC
CATCGCTGACTAATTTTTTTGCAGAGGCCGTTTTTTTTTTTTTTTAGGCCGC
CTCGGCCTCTGAGCTTTTTTTTTTTTATTCCAGAA**TATATAT**GTAGTGAG
CAGGCTTTTTTGGCGACCTAGGCTT

MLPcore
TATAAAAGGGGGTGGGGGCGTTCGTCCTCACTCT

pCMVcore
TATATAAGCA<u>C</u>AGCTCGTTTAGTGAACCG**T**CAGATCGCCTG

pNMT1core

TATATAAAGGAAGAGGAATCCTGGCATA**A**TCATCAATTGAATAAGTTGAAT
TAATTATTTCAATCTCATTCTCACTTTCTGACTTATAGTCGCTTTGTTAAAT
GGCC

pJB42CAT5core

TATAAAAGCTTGGGGCTGGGGCCGAGCAC**T**GGGGACTTTGAGGGTGGCC
AGGCCAGCGTAGGAGGCCAGCGTAcGATCCTG

pCaMV35Score

CAAGACCCTTCCTC**TATATAA**GGAAGTTCATTTCATTTGGAGAGGA

pTKcore

ATATTAAGGTGACGCGTGTGGCCTCGAACACCGAGCGACCCTGCAGCGAC
CCGCTTAA

UAS$_{GPD(long)}$

CAGTTCGAGTTTATCATTATCAATACTGCCATTTCAAAGAATACGTAAATA
ATTAATAGTAGTGATTTTCCTAACTTTATTTAGTCAAAAAATTAGCCTTTT
AATTCTGCTGTAACCCGTACATGCCCAAAATAGGGGGCGGGTTACACAGA
ATATATAACATCGTAGGTGTCTGGGTGAACAGTTTATTCCTGGCATCCACT
AAATATAATGGAGCCCGCTTTTTAAGCTGGCATCCAGAAAAAAAAAGAAT
CCCAGCACCAAAATATTGTTTTCTTCACCAACCATCAGTTCATAGGTCCAT
TCTCTTAGCGCAACTACAGAGAACAGGGGCACAAACAGGCAAAAAACGG
GCACAACCTCAATGGAGTGATGCAACCTGCCTGGAGTAAATGATGACACA
AGGCAATTGACCCACGCATGTATCTATCTCATTTTCTTACACCTTCTATTA
CCTTCTGCTCTCTCTGATTTGGAAAAAGCTGAAAAAAAAGGTTGAAACCA
GTTCCCTGAAATTATTCCCCTACTTGAC

UAS$_{GPD(40nt)}$

GTAGGTGTCTGGGTGAACAGTTTATTCCTGGCATCCACTA

UAS$_{TEF2(59nt)}$

AAACTACCTCCGTACATTCATGTTGCACCCACACATTTATACACCCAGACC
GCGACAAA

UAS$_{FEC}$

CCTCCTTGAAACTGAAATTTTAGCATGTGA

$\mathrm{UAS}_{\mathrm{TEF1(long)}}$

ATAGCTTCAAAATGTTTCTACTCCTTTTTTACTCTTCCAGATTTTCTCGGAC
TCCGCGCATCGCCGTACCACTTCAAAACACCCAAGCACAGCATACTAAAT
TTCCCCTCTTTCTTCCTCTAGGGTGTCGTTAATTACCCGTACTAAAGGTTTG
GAAAAGAAAAAAGAGACCGCCTCGTTTCTTTTTCTTCGTCGAAAAAGGCA
ATAAAAATTTT

$\mathrm{UAS}_{\mathrm{TEF1(RAP1bs)}}$

CTTCAA**aaCACCCAAGCACag**CATACt

FIGURES

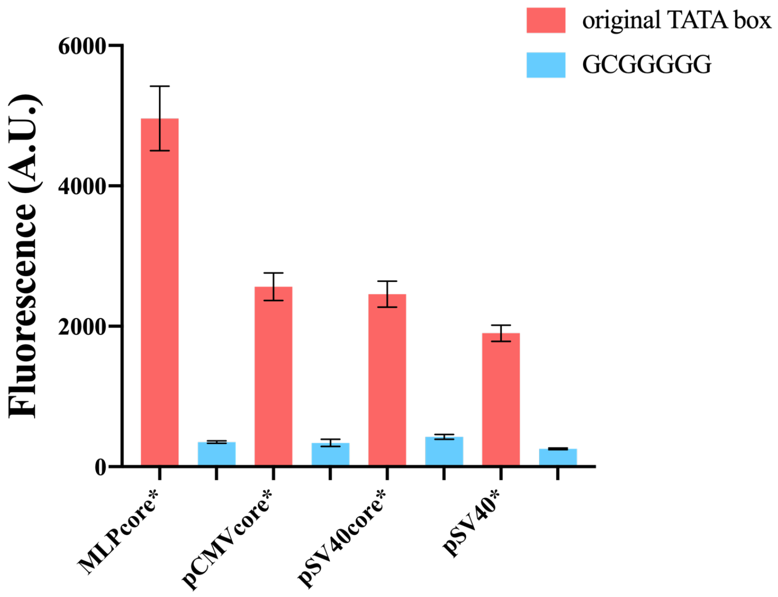

Figure S1. Reduction in the mean fluorescence expression due to the substitution of the original TATA box, of four of our new synthetic promoters, with the heptamer GCGGGGG. The mean fluorescence values in the presence of GCGGGGG are (in A.U.): 349.94 (MLPcore*); 339.91 (pCMVcore*); 425.28 (pSV40core*); and 253.65 (pSV40*). It is reasonable to assume that they are due to pCYC1noTATA, whose mean fluorescence amounts to 343.64 A.U. (see Table S1) and can be attributed to leakage. Each average value comes from three independent experiments.

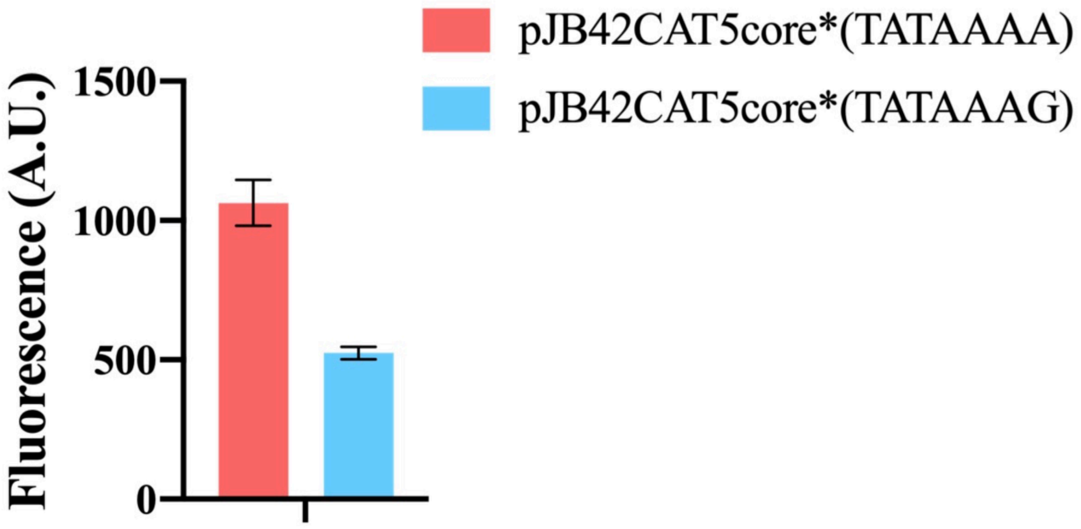

Figure S2. Effect on the mean fluorescence, expressed by the synthetic core promoter pJB42CAT5core*, due to a G → A mutation at position 7 of the TATA box. Each average value comes from three independent experiments.

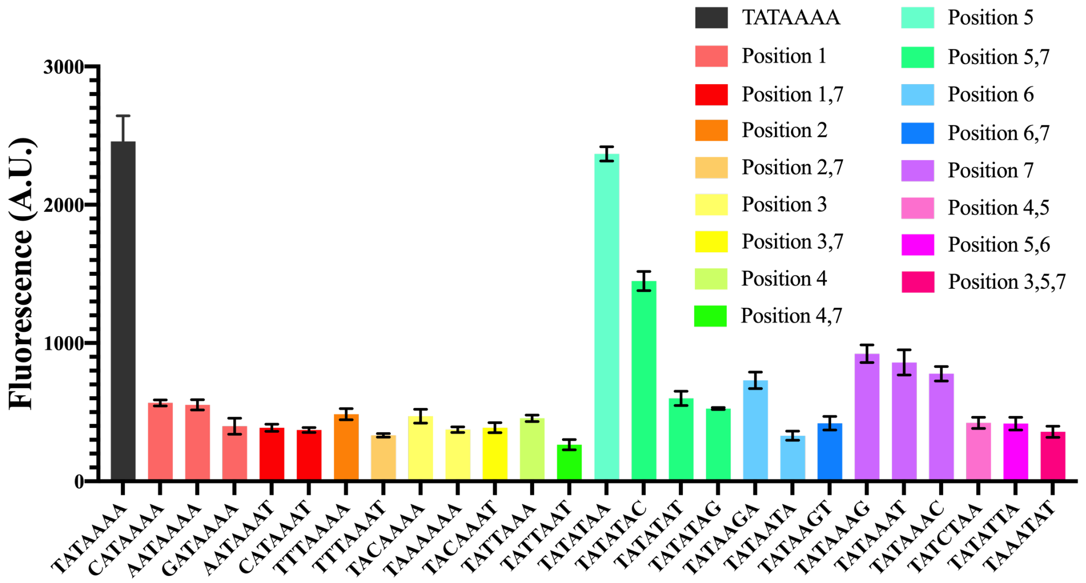

Figure S3. Variation in the mean fluorescence expressed from the synthetic pSV40core* due to one or multiple mutations along the strong TATA box—TATAAAA. Each average value comes from three independent experiments.

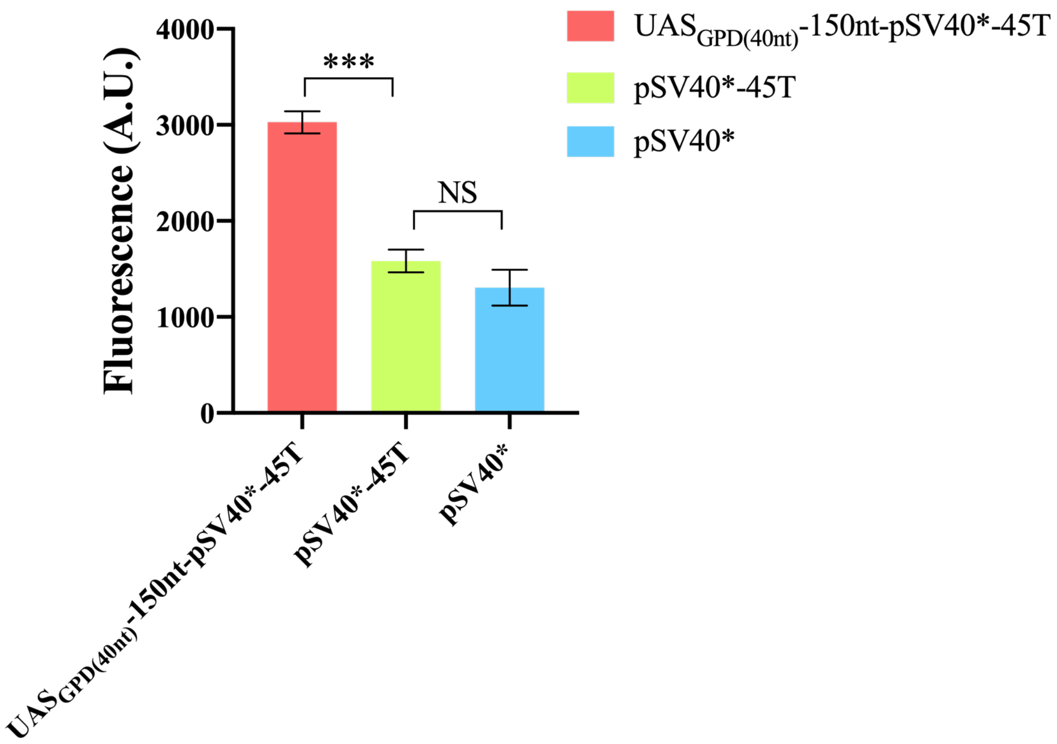

Figure S4. Mean fluorescence expressed by pSV40* variant containing three islands of 15 thymines each for nucleosome removal. Only the addition of the strong $UAS_{GPD(40nt)}$ 150 nt upstream of the TATA box contributed to an enhancement in the promoter strength. The variant of pSV40*-45T showed no significant difference in statistical terms from the original pSV40* (NS, p-value = 0.1096). "***" corresponds to a p-value < 0.001, two-sided Welch's t-test.
